# Mr. Guthrie on Oleum Terebinthinæ in Internal Inflammation of the Eye

**Published:** 1830-01-01

**Authors:** 


### XXXVIII.

### ROYAL WESTMINSTER OPHTHALMIC HOSPITAL.

Mr. Guthrie has continued the trial of the oleum terebinthinæ in several cases of internal inflammation of the eye, in some cases of amaurosis, and in other complaints of an anomalous nature. We have selected the four following of internal inflammation.

*Case* 1. Daniel Kelly, ætatis 26, admitted Sept. 1st, 1829, with iritis of the right eye.

Two years ago the left eye was affected with the same disease for which he was under the care of Mr. Guthrie, and was cured by calomel. The inflammation of the right eye commenced four days ago with slight pains in the orbit, which he has not felt for the two last days: the iris is discolored, and the pupil irregular; the sclerotic vessels are enlarged forming a zone of a pink color round the circumference of the cornea, the vessels of the conjunctiva are also enlarged.

2nd, The inflammation is much increased, the iris being of a darker color, and the pupil more irregular; the zone round the cornea is much more distinct and of a darker color, he complains of the tears being very hot.

Ol. terebinth. ʒj. ter quotidie.

3d, The pupil is contracted and acts very sluggishly; the conjunctiva more vascular, the tears continue hot, and he complains of slight pain in the eye on exposure to light; he voids his water more frequently and has a little scalding.

Cont. ol. terebinth. ʒj. ter die.

5th, He suffered great pain in the eye last night. The iris is more discolored, and the sclerotic inflammation augmented; the vessels of the conjunctiva are less injected.

App. Cucurbit. cum ferro ad tempor. ad ℥x.

Cont. ol. terebinth. ʒiss. ter die.

8th, The sclerotica less inflamed, and the conjunctival inflammation quite gone; the iris of a lighter color; the pupil remains contracted and irregular: he has not taken any medicine since the 6th. Ordered to leave off taking the ol. terebinth. and to have the guttæ belladonnæ dropped into the eye.

15th, The sclerotic inflammation almost gone, and the pupil nearly circular, and more dilated.

App. guttæ belladonnæ.

17th, The pupil is now quite regular, and the iris is of the same color as that of the other eye, and the redness of the sclerotica has quite disappeared.

Discharged cured.

Case kept by Mr. Weight.

*Case* 2. Mary Barry, æt. 40, admitted August 28th, 1829. About a fortnight ago was attacked by severe darting pains in the left eye-ball, accompanied by inflammation which she attributes to cold. She was cupped to ℥xiij. the next week and purged. The iris is now slightly discolored; pupil fixed and irregular; sclerotica and conjunctiva a good deal inflamed; cornea rather muddy; she complains of a feeling as if sand were between the eye-lid and the ball of the eye.

Cap. ol. terebinth. ʒj. ter die.

29th, The sclerotic and conjunctival inflammation is considerably diminished. The first dose of turpentine made her very sick. Rep.

Sept. 3rd, Sclerotic inflammation nearly gone; pupil more regular; the cornea is still muddy, and she says that her sight is more dim. Complains of a little strangury.

Pulv. jalap, c. ʒj.

Infus. lini. p. p. v.

Cont. ol. terebinth.

10th, Has not taken any turpentine the last three days. She is however much better—pupil contracts and dilates and is almost regular; sight improved—bowels confined.

Pulv. jalap, c. ʒj. o. n.

Gutt. belladonnæ.

17th, All appearances of inflammation are gone, and she only complains of a little dimness of sight.

Gutt. belladonnæ.

Sept. 24th. Has returned to-day suffering under a fresh attack of the disease. attributes it to her getting wet on the 19th. The iris is very irregular, darker in color than natural and immoveable; cornea muddy; sclerotica and conjunctiva slightly inflamed; complains of darting pains in the eye.

Ol. terebinth. ʒj. ter die.

Oct. 6th. Has taken the medicine pretty regularly since the 24th, and excepting a little dimness of vision she is quite well.

Gutt. belladonnæ.

Case kept by Mr. James.

*Case* 3. John Lucas, aged 20, admitted Sept. 22d, having iritis of both eyes. Inflammation attacked the right eye a fortnight ago, and the left three days after. He complains now of great pain across his forehead and intolerance of light, his sight he says is very dim, especially of the left eye. He applied a blister behind his left ear three days back, but without any relief. The conjunctiva of both eyes is very much inflamed so that the state of the sclerotica cannot be seen: the aqueous humour very turbid, and particularly so in the *right* eye which he says he can see best with; iris in both eyes very irregular and immoveable. There is a lichenous eruption on his body which he says he first observed three months ago; it is unaccompanied by sore throat or other constitutional symptoms. Says that he had a gonorrhœa two years ago, but he denies ever having had a chancre.

Cap. ol. terebinth. ʒj. ter die.

23d, He only took one dose of the turpentine yesterday; the pain however is very much diminished, bowels open.

Cont. med.

24th, Took his three doses regularly yesterday. Says that he suffers no pain now, and that his sight has very much improved; the aqueous humour in both eyes is evidently clearer, the redness remains as yet uninfluenced by the medicine. Rep.

25th, The pain has returned with increased violence—inflammation of the conjunctiva much worse, and he complains of the discharge of hot tears: took the turpentine regularly yesterday, bowels open; no strangury or increased flow of urine. To lose eight ounces of blood from each temple immediately and to continue his medicine.

Pulv. jalap, c. ʒj.

26th, Much better; pain quite gone; the inflammation of conjunctiva is very acute, and he says that his sight is not so clear to-day. To apply six leeches to the lower lid of each eye and to continue his medicine.

27th, Only applied three leeches to each eye; the inflammation is however a little diminished. To repeat the leeches to-day and to continue taking his medicine.

29th, Complains to-day of a little pain in his right eye; inflammation of conjunctiva diminished; aqueous humour clearer; pupils remain very irregular and fixed.

Gutt. belladonnæ.

Cont. ol. terebinth.

Oct. 1st, Has taken the turpentine regularly, but complains that it gives him pain when making water, bowels open. The inflammation of both eyes remains much the same.

Omittr. ol. terebinth.

Pulv. jalap, c ʒj.

Rep. gutt. bellad.

8th, Has not taken any of the turpentine since the 1st: the drop of belladonna has been applied regularly to each eye every morning, and his bowels have been freely acted upon by the compound jalap powder, all pain in making water has ceased; the inflammation in both eyes is considerably diminished and he can see better. The lichenous eruption is much thicker all over his body.

Cap. ol. terebinth. ʒj. ter die.

Rep. belladonna.

11th, The left eye is better; sclerotic inflammation diminished, pupil rather more contracted but nearly circular, aqueous humour clearer. The right eye is worse, conjunctival and sclerotic inflammation a good deal increased, and the iris has two small elevated spots on its surface; sight more dim; does not complain of any pain but a sensation of heat in this eye. The tupentine causes strangury, but not to that degree to prevent him continuing to take it. To apply six leeches to the right eye.

Pulv. jalap. comp.

Infus. lini, p. p.

13th, Very little inflammation remaining in the left eye; but in the right it is much the same, pupils of both more dilated and regular. Takes the turpentine regularly now without any unpleasant effects. Rep. med.

20th, Has taken his medicine regularly since the last report. The left eye is now quite well excepting a slight irregularity of pupil. The inflammation of the right eye is also considerably diminished; the eruption has disappeared in some degree about the arms, but remains very plentiful on his back.

Cont. ol. terebinth.
Rep. belladonna.

Nov. 3d, To-day the ung. argent. nit. is applied to the right eye to relieve the chronic turgescence of the vessels which appears to be all that remains of the disease.

Omittr. ol. terebinth.

5th, No redness whatever remaining; the pupil is however rather irregular. Gutt. belladonnæ.

10th, Returned to-day with slight inflammation of the conjunctiva of both eyes. Ung. arg. nit.

20th, Since last report he has not had any complaint.

Case kept by Mr. Taylor.

*Case* 4. *Syphilitic Iritis treated by the Oleum Terebinthinæ.*—William Anderson, æt. 37, admitted Nov. 5th, 1829, states, that about a twelvemonth ago he had sores on the penis and suppurating buboes, for which he was salivated in the Winchester Hospital. About three months after, a lichen appeared on the arms, sometimes disappearing, but not entirely, and always re-appearing in the course of a month. He has at present a few spots remaining on the arms, shoulders, and legs, also a large crop on the back of the neck—has had a sore throat for the last three months. The iritis is of the right eye and has existed about a week. The iris is changed in color and irregular—is also much contracted, and does not obey the stimulus of light. The sclerotic inflammation is intense, extending to the conjunctiva of the lids—vision is nearly lost—complains of great pain round the brow, and a sense of soreness in the ball of the eye, attended with great lacrymation.

Mist. terebinth. ℥j. ter in die.

7th, Iris irregular—more changed in color—anterior chamber rather cloudy: complains of having suffered severe pain in the brow last night, which lasted three or four hours.

Cucurb. cruent. ad ℥xij. temp.
Mist. rep. ter in die.

8th, The cupping has removed the pain—says he thinks he sees better—iris still much contracted and irregular—color not so much changed—sclerotic inflammation much relieved. Says that he does not feel any nausea, but that he has great pain in the penis on making water. Bowels confined.

Pulv. jalap. c. ʒiss. statim.
Mist. rep. ter in die.—App. gutt. bellad. ad ocul.
Dec. hordei ad libitum—p. potu ordinario.

9th, Mist. rep.—Dec. hordei.

10th, Pupil dilates a little—is rather irregular—iris slightly changed in color—sclerotic inflammation is nearly removed—is entirely free from pain in either eye or head—complains much of strangury, having severe pain in the whole course of the urethra, after the discharge of some bloody urine, which occurs every half hour.

Omitte mist. terebinth.
Pulv. jalap, c. ʒiss. statim.
Gutt. bellad.
Cont. dec. hordei.

11th, Iris has regained its original colour—pupil very nearly regular—sees a great deal better—there is still some slight sclerotic inflammation—eruption appears much the same—complains severely of the strangury—states that it prevents his sleeping during the whole night, being obliged to rise every ten minutes. The powder had not any effect.

Cucurb. cruent. ad. ℥xij.—à perineo.

Hyd. submur. gr. vj. h. s. s.

Pulv. rhei. ʒss. mane sumend.

Gutt. bellad.—cont. dec. hordei.

12th, Sees a great deal better—has not had any terebinthina for the last two days on account of the irritation it produced—was ordered to be cupped yesterday on the perineum, but was cupped on the temples by mistake—strangury still severe.

Gutt. bellad. Decoct. hordei ad libitum.

14th, Iris is again much changed in color, with contracted pupil—sclerotic inflammation very severe—vision as imperfect as in the commencement of the complaint—complains much of the strangury—has bloody and painful micturitions every hour.

Hyd. subm. gr. vj. h. s. s. Magn. sulph. ℥j. mane.
Cont. dec. hordei.

15th, Is free from pain in the eye—thinks he sees a little better—sclerotic inflammation not so severe—iris still much changed in color, with irregular pupil—has less pain after making water, and he does not pass so much blood.

Pil. rep. et magn. sulph. Dec. hordei.
Gutt. belladonnæ.

17th, Is much improved—says that he sees much better, and his eye feels stronger, and free from pain—strangury much less severe.

Gutt. bellad.

19th, The iris has regained its proper color—signs of inflammation are gone, and all the pupil still remains partly irregular—vision a little imperfect—strangury nearly gone.

Gutt. belladonnæ.

Case kept by Mr. Foot.

